# An Efficient and Recyclable Nanoparticle-Supported Cobalt Catalyst for Quinoxaline Synthesis

**DOI:** 10.3390/molecules201119731

**Published:** 2015-11-19

**Authors:** Fatemeh Rajabi, Diego Alves, Rafael Luque

**Affiliations:** 1 Department of Science, Payame Noor University, P. O. Box: 19395-4697, Tehran 19569, Iran; 2 Laboratório de Síntese Orgânica Limpa—LASOL, Universidade Federal de Pelotas UFPEL, Pelotas, CEP 96010-900, Brazil; diego.alves@ufpel.edu.br; 3 Departamento de Quimica Organica, Universidad de Cordoba, Campus de Rabanales, Edificio Marie Curie (C3), Ctra Nnal IV-A, Km 396, Cordoba E14014, Spain; q62alsor@uco.es

**Keywords:** quinoxaline, catalysis, nanoparticles, cobalt, green chemistry

## Abstract

The syntheses of quinoxalines derived from 1,2-diamine and 1,2-dicarbonyl compounds under mild reaction conditions was carried out using a nanoparticle-supported cobalt catalyst. The supported nanocatalyst exhibited excellent activity and stability and it could be reused for at least ten times without any loss of activity. No cobalt contamination could be detected in the products by AAS measurements, pointing to the excellent activity and stability of the Co nanomaterial.

## 1. Introduction

**Figure 1:**
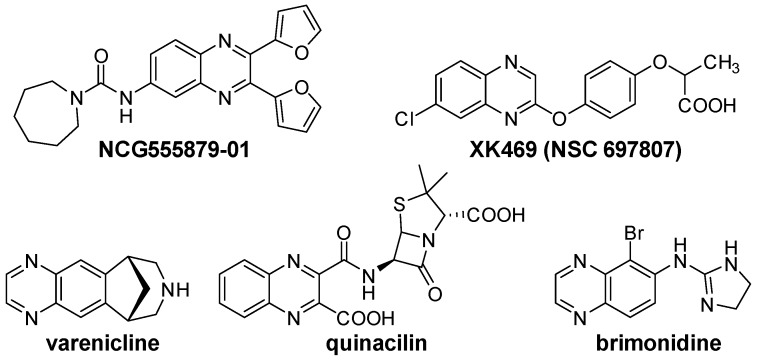
Biologically important quinoxalines.

Quinoxaline derivatives are attractive *N*-containing heterocycles and these scaffolds have attracted much attention, not only in synthetic chemistry [1,2,3] but also in the medicinal field [4,5,6,7,8,9,10,11]. These compounds exhibit diverse biological activities, such as antiviral [4,5], antibacterial [6], anti-inflammatory [7], antitumoral [8,9] and anti-HIV properties [10,11]. Examples of quinoxaline- containing pharmacological entities are shown in Figure 1. In addition, quinoxalines have been applied as building blocks for the development of macrocyclic molecular receptors [12,13], semiconducting materials [14,15,16,17,18,19,20], dyes [21], cavitands [22] and luminescent materials [23].

Generally, quinoxalines can be prepared via a double condensation of 1,2-phenylenediamines with 1,2-diketones [24,25,26,27,28]. A number of reagents have been shown to catalyze these reactions such as acidic alumina [29], citric acid [30], magnetic Fe_3_O_4_ nanoparticles in H_2_O [31], silica-bonded sulfonic acid [32], among others [33,34]. Other protocols to synthesize quinoxalines mainly involve the oxidative trapping of vicinal diols or α-hydroxy ketones with 1,2-diamines [35,36,37,38,39,40,41,42], 1,4-addition of 1,2-diamines to diazenylbutenes [43], coupling of epoxides with ene-1,2-diamines [44,45], 2-nitroanilines with phenethylamines [46], alkynes or ketones with 1,2-diamines via a key oxidation process [47,48,49,50,51]. Therefore, the development of efficient methods for accessing quinoxalines derivatives continues to be an active area of research.

Nanoparticle-supported catalysts can offer important advantages as compared to homogeneous transition metal systems and colloidal nanoparticles. These include a good reusability coupled with high activities and specificities in different chemistries based on their excelling properties (high surface areas, degenerated density of energy states and plasmon) [52,53,54]. In this regard, Co/supported catalysts were previously reported to be highly active and versatile for acid and redox catalyzed processes [54,55].

**Scheme 1:**
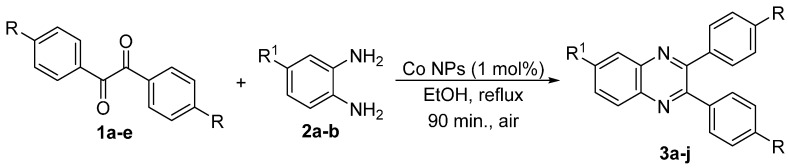
General scheme of the reactions.

To the best of our knowledge, there is no protocol describing the preparation of quinoxaline derivatives using a nanoparticle-supported cobalt catalyst. In view of the explained above, we decided to examine the synthesis of substituted quinoxalines by reaction of 1,2-diketones with 1,2-phenylenediamines using a nanoparticle-supported cobalt catalyst (Scheme 1).

## 2. Results and Discussion

Initially, we chose 1,2-diphenylethanedione (**1a**)and 1,2-diamino-4-nitrobenzene (**2a**) as model substrates to establish the best conditions for this reaction and some experiments were performed to synthesize the corresponding quinoxaline **3a** (Table 1). We started our studies reacting 1,2-diketone **1a** (1.0 mmol) with 1,2-phenylenediamine **2a** (1.0 mmol) at 100 °C for 2 h, without catalyst and solvent. Under these conditions, product **3a** was not obtained (Table 1, entry 1). Good results were obtained however when the reactions of substrates **1a** and **2a** were carried out using H_2_O as solvent in the presence of Co NPs (2 mol %) as catalyst. Reactions performed at 100 °C and 50 °C gave the desired product in 87% and 57% yield, respectively (Table 1, entries 2 and 3). A similar result was obtained when the reaction was conducted at 100 °C, however using 1 mol% of Co NPs (86% yield) (Table 1, entry 4). Good results were also found when the reactions were performed using EtOH as solvent (Table 1, entry 5–9). Excellent yields of product **3a** were achieved in reactions carried out in EtOH at 78 °C using 1 mol% of catalyst (Table 1, entries 7–8). When the amount of catalyst was reduced to 0.5 mol %, a decrease in the yield of product **3a** was observed (Table 1, entry 9). Finally, the reaction performed using 1 mol % of Co NPs at 100 °C and in absence of EtOH yielded the quinoxaline **3a** in 72% yield (Table 1, entry 10).

Analyzing the results shown in Table 1, we established the best reaction conditions reacting 1,2-diphenylethanedione (**1a**, 1.0 mmol, 0.033 g) with 1,2-diamino-4-nitrobenzene (**2a**, 1.0 mmol) using supported CoNPs (1 mol %) as catalyst and EtOH (5 mL) as solvent. After that, the mixture was stirred at reflux for 90 min in open atmosphere, affording 6-nitro-2,3-diphenylquinoxaline(**3a**) in 92% yield after crystallization.

**Table 1:** Optimization of reaction condition ^a^.
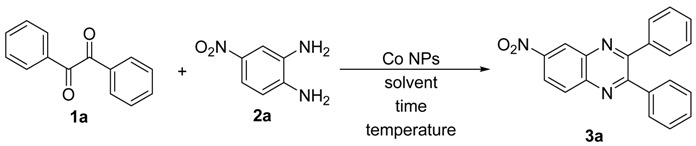

Entry	Catalyst (mol %)	Solvent	Temperature (°C)	Time (h)	Yield 3a (%) ^b^
1	-	-	100	2	-
2	2	H_2_O	100	2	87
3	2	H_2_O	50	2	57
4	1	H_2_O	100	2	86
5	2	EtOH	78	2	93
6	2	EtOH	50	2	60
7	1	EtOH	78	2	93
**8**	**1**	**EtOH**	**78**	**1.5**	**92**
9	0.5	EtOH	78	1.5	80
10	1	-	100	2	72

^a^ Reactions are performed using, 1,2-diphenylethanedione **1a** (1.0 mmol) and 1,2-diamino-4-nitrobenzene **2a** (1.0 mmol) in open atmosphere. ^b^ Yields are given for isolated product **3a** after crystallization.

In order to extend the scope of the reaction, the best conditions were employed in reactions of 1,2-diamino-4-nitrobenzene (**2a**) with other 1,2-diketones **1b**–**e** with different patterns of substitution and the results are summarized in Table 2. As it can be seen on Table 2 (Entries 1–5), our methodology is suitable to a range of substituted 1,2-diketones containing electron-withdrawing groups, affording excellent yields to desired products in all examples. In addition, the possibility of performing the reaction of 1,2‑diketones **1a**–**e** with *o*-phenylenediamine (**2b**) was also investigated (Table 2, entries 6–10). Using these substrates, a range of substituted quinoxalines was obtained in excellent yields using the nanoparticle-supported cobalt catalyst under optimized reaction conditions.

**Table 2:** Generality of the reaction of 1,2-diketones with 1,2-diamines ^a^.
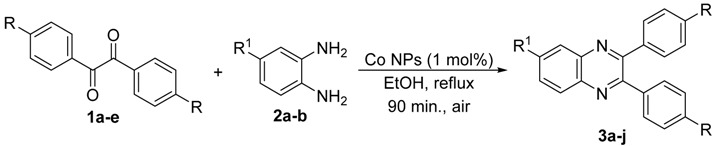

Entry	1,2-Diketone 1	1,2-Diamines 2	Product 3	Yield (%) ^b^	M.P. (°C)
1	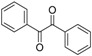 **1a**	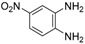 **2a**	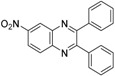 **3a**	92	188–190
2	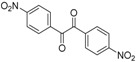 **1b**	**2a**	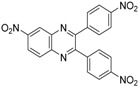 **3b**	92	195–197
3	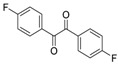 **1c**	**2a**	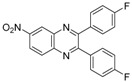 **3c**	92	173–175
4	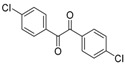 **1d**	**2a**	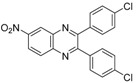 **3d**	90	175–177
5	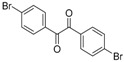 **1e**	**2a**	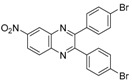 **3e**	88	143–145
6	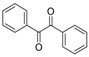 **1a**	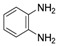 **2b**	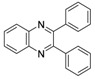 **3f**	92	127–129
7	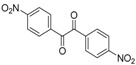 **1b**	**2b**	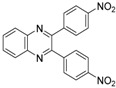 **3g**	96	127–129
8	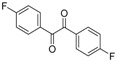 **1c**	**2b**	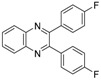 **3h**	94	133–135
9	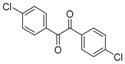 **1d**	**2b**	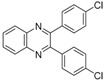 **3i**	94	190–192
10	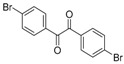 **1e**	**2b**	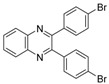 **3j**	94	134–135
11	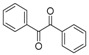	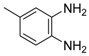 **2c**	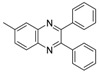 **3k**	98	116–117
12	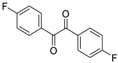	**2c**	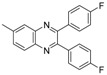 **3l**	95	163–165

^a^ Reactions were performed using 1,2-diketones **1a**–**e** (1.0 mmol), 1,2-diamines **2a**–**b** (1.0 mmol), supported CoNPs (1 mol %, 0.033 g) and EtOH (5 mL) at reflux in open flask for 90 min. ^b^ Yields are given for isolated products after crystallization.

Reused runs were carried out under similarly optimized conditions using 5 mmol 1,2-diphenylethanedione (**1a**), 5 mmol of 1,2-diamino-4-nitrobenzene (**2a**) and supported cobalt catalyst (0.05 mmol, 0.165 g) at 78 °C in 10 mL of ethanol. The catalyst showed excellent recoverability and reusability over ten successive runs under the same conditions as the first run. It is quite remarkable that all materials discussed in this study exhibited outstanding structural stability by TGA (results not shown). The cobalt catalyst was found to be highly stable and reusable under the investigated conditions (up to 12 runs) without any significant loss of its catalytic activity (Table 3). Indeed, ICP analysis of both reaction filtrate and catalyst showed no detectable Co leaching (<0.5 ppm) in the reaction filtrate upon reaction completion, with an almost identical Co content for both fresh and reused catalyst (0.30 *vs.* 0.29 mmol of Co per gram of catalyst for fresh and 10-time reused material, respectively).

**Table 3:** Reuses of the supported CoNP catalyst in the reaction of 1,2-diphenylethanedione (**1a**) with 1,2-diamino-4-nitrobenzene (**2a**).

Run No. ^a^	1	2	3	4	5	6	7	8	9	10
Yield (%) ^b^	94	94	92	92	92	90	91	90	90	87

^a^ Reaction conditions: 1,2-diphenylethanedione (5.0 mmol) and 1,2-diamino-4-nitrobenzene (5.0 mmol), supported CoNPs ( 0.05 mmol, 0.165 g) in EtOH (10 mL) at reflux conditions for 90 min. ^b^ Isolated yields.

The study of the scale-up reaction (from 1 to 20 mmol of substrate) was also investigated under the optimized reaction conditions. When the amount of **1a** and **2a** was increased to 20 mmol, the same conversion was obtained after 90 min under optimized conditions.

The catalytic performance of our system was eventually compared to reported literature data. As can be seen in Table 4, our recoverable catalytic system possesses remarkably improved activities as compared to those of related previously reported heterogeneous systems.

**Table 4:** Comparison of the result in the reaction of 1,2-diphenylethanedione (**1a**) with 1,2-diaminobenzene (**2a**) with our method and the previous literature.

Entry	Condition	Time (min)	Yield (%)	Reference
1	Polyaniline-sulfate salt (5 wt %), DCE, r.t.	20	95	[25]
2	CAN (5 mol %), H_2_O, r.t.	10	98	[26]
3	I_2_ (10 mol %), DMSO, r.t.	35	95	[27]
4	MeOH:AcOH (9:1), MW, 160 °C	5	99	[28]
5	Acidic alumina, 80 °C	2	96	[29]
6	Citric acid (10 mol %), EtOH, r.t.	1	94	[30]
7	Fe_3_O_4_NPs (10 mol %), H_2_O, r.t.	150	95	[31]
8	Silicabonded *S*-sulfonicacid (3.4 mol %), EtOH/H_2_O (70/30), r.t.	5	96	[32]
9	Ga(OTf)_3_ (1 mol %), EtOH, r.t.	5	99	[33]
10	Bi(OTf)_3_ (10 mol %), H_2_O, r.t.	5	97	[34]
11	CoNP (1mol %), EtOH, reflux	90	92	Our work

## 3. Experimental Section

### 3.1. General Information

Unless otherwise stated, all reagents and chemicals in this study were used as received and were not further purified (Sigma-Aldrich Chemie GmbH, Taufkirchen, Germany). Melting point recorded on a RY-1 microscopic melting apparatus (Hangzhou Chincan Trading Co., Shanghai, China) and uncorrected. ^1^H-NMR and ^13^C-NMR spectra were respectively recorded on 500 MHz and 125 MHz by using a Bruker Avance 500 spectrometer (Bruker BioSpin GmbH, Rheinstetten, Germany). Metal content in the materials was determined using inductively coupled plasma (ICP) in a Philips PU 70000 sequential spectrometer (Philips, Almelo, The Netherlands) equipped with an Echelle monochromator (0.0075 nm resolution). Samples were digested in HNO_3_ and subsequently analyzed by ICP Nitrogen adsorption measurements (Philips) were carried out at 77 K using an ASAP 2000 volumetric adsorption analyzer from Micromeritics (Micromeritics, Norcross, GA, USA). The samples were outgassed for 24 h at 100 °C under vacuum (p b 10–2 Pa) and subsequently analyzed.

### 3.2. Preparation of the Supported Cobalt Catalyst

CoNPs was synthesized as previously reported [55]. Briefly, salicylaldehyde (2 mmol, 0.244 g) was added to an excess of absolute MeOH, to which 3‑aminopropyl(trimethoxy)silane (2 mmol, 0.352 g) was subsequently added. The color of the solution instantly changed to yellow indicating imine formation. After 3 h, cobalt (II) acetate, Co(OAc)_2_·2H_2_O (1 mmol, 0.248 g) was added to the solution, and the mixture stirred for three additional hours to allow the new ligands to complex the cobalt. A color change from pink to olive green is observed. SBA-15 (3 g) was activated by refluxing in concentrated hydrochloric acid (6 M) and then washed thoroughly with deionized water and dried before undergoing chemical surface modification. This activation treatment readily hydrolyses the siloxane Si-O-Si bonds to Si-OH species which will be key to anchor the cobalt complex. Both complex and activated silica were then mixed and the mixture was stirred overnight. The solvent was removed using a rotary evaporator, and the resulting olive green solid dried at 80 °C overnight. The final product was washed with MeOH and water (to remove all physisorbed metal species) until the washings were colourless. Further drying of the solid product was carried out in an oven at 80 °C for 8 h. The loading of cobalt was calculated about 0.3 mmol·g^−1^ and surface analysis showed cobalt oxide species well dispersed on the surface of SBA-15 with 450 m^2^·g^−1^ surface area and pore size of 3.6 nm with 0.77 cm^3^·g^−1^ mesoporous pore volume.

### 3.3. General Reaction Procedure 

To a mixture of 1,2-dicarbonyl compound **1a**–**e** (1.0 mmol) and 1,2-diamine **2a**–**b** (1.0 mmol) in ethanol (5 mL), supported CoNP (0.033g, 1 mol%) was added and the mixture was refluxed in an open flask for 90 min. Reactions were monitored by thin layer chromatography (TLC) until total disappearance of the starting material. After completion of the reaction, the reaction mixture was cooled to room temperature, and resulting solid was collected by filtration and dissolved in ethyl acetate (10 mL). The supported catalyst was recovered by filtration. After evaporation of solvent, the resulting solid product was purified by crystallization in ethanol.

### 3.4. Selected Spectroscopic Data 

*6-Nitro-2,3-diphenylquinoxaline* (Table 2, Entry 1, **3a**). Yellow solid; m.p. 188–190 °C (lit. [56] 193–194 °C). ^1^H-NMR (CDCl_3_): δ 7.38 (m, 6H, Ar-H), 7.56 (m, 4H, Ar-H), 8.28 (m, 1H, Ar-H), 8.45 (m, 1H, Ar-H), 9.02 (m, 1H, Ar-H); ^13^C-NMR (CDCl_3_): δ 123.27, 125.51, 128.45, 129.67, 129.85, 129.95, 130.66, 137.95, 139.87, 143.39, 147.80, 155.62, 156.18.

*2,3-Diphenylquinoxaline* (Table 2, Entry 6, **3f**). White solid; m.p. 127–129 °C (lit. [26] 126–127 °C). ^1^H-NMR (CDCl_3_): δ 7.35 (m, 6H, Ar-H), 7.56 (m, 4H, Ar-H), 7.76 (m, 2H, Ar-H), 8.20 (m, 2H, Ar-H); ^13^C-NMR (CDCl_3_): δ 128.29, 128.89, 129.13, 129.915, 130.10, 138.92, 141.15, 154.38.

*2,3-Bis(4-Fuorophenyl)quinoxaline* (Table 2, Entry 8, **3h**). White solid; m.p. 133–135 °C (lit. [56] 135–137 °C). ^1^H-NMR (CDCl_3_): δ 7.06 (m, 4H, Ar-H), 7.52 (m, 4H, Ar-H), 7.80 (q, *J* = 9.5 Hz, 1H, Ar-H), 8.16 (q, *J* = 9.1 Hz, 1H, Ar-H); ^13^C-NMR (CDCl_3_): δ 115.45, 115.61, 129.14, 130.22, 131.70, 131.82, 134.90, 135.02, 141.21, 152.16, 161.54, 164.80.

*2,3-Bis(4-Chlorophenyl)quinoxaline* (Table 2, Entry 9, **3i**).White solid; m.p. 190–192 °C (lit. [32] 195–196 °C). ^1^H-NMR (CDCl_3_): δ 7.32 (m, 4H, Ar-H), 7.49 (m, 4H, Ar-H), 7.72 (m, 2H, Ar-H), 8.11 (m, 2H, Ar-H); ^13^C-NMR (CDCl_3_): δ 128.50, 128.62, 129.05, 129.11, 129.17, 130.05, 130.13, 130.26, 131.30, 134.12, 137.36, 138.62, 140.09, 141.11, 153.02, 153.18.

*6-Methyl-2,3-diphenylquinoxaline* (Table 2, Entry 11, **3k**). Brown solid; m.p. 116–118 °C (lit. [33] 117–118 °C). ^1^H-NMR (CDCl_3_): δ 2.61 (s, 3H, Ar-CH_3_ ), 7.35 (s, 6H, Ar-H), 7.55 (d, *J* = 6.5, 4H, Ar-H), 7.60 (s, 1H, Ar-H) 7.98 (s, 1H, Ar-H), 8.09 (d, *J* = 8.4, 1H, Ar-H); ^13^C-NMR (CDCl_3_): δ 21.95, 128.04, 128.24, 128.67, 128.73, 129.90, 129.92, 132.32, 139.24, 139.73, 140.49, 141.29, 152.55, 153.29.

## 4. Conclusions

In summary, we have developed an environmentally friendly and highly active cobalt nanoparticle on mesoporous SBA-15 material for the synthesis of quinoxalinesin excellent yields from 1,2-diamine and 1,2-dicarbonyl compounds. Reactions could efficiently afford the target products after short reaction times and were run under air and mild reaction conditions and require low loadings of the supported catalyst. The catalyst was found to be highly reusable for at least ten reaction runs under the investigated conditions.
